# Non-Extensive Statistical Analysis of Acoustic Emissions Recorded in Marble and Cement Mortar Specimens Under Mechanical Load Until Fracture

**DOI:** 10.3390/e22101115

**Published:** 2020-10-02

**Authors:** Andronikos Loukidis, Dimos Triantis, Ilias Stavrakas

**Affiliations:** Electronic Devices and Materials Laboratory, Department of Electrical and Electronics Engineering, University of West Attica, 250 Thivon Avenue, 122 44 Athens, Greece; triantis@uniwa.gr (D.T.); ilias@uniwa.gr (I.S.)

**Keywords:** non-extensive statistical physics, Tsallis entropy, q-exponential distribution, acoustic emissions, marble, cement

## Abstract

Non-extensive statistical mechanics (NESM), which is a generalization of the traditional Boltzmann-Gibbs statistics, constitutes a theoretical and analytical tool for investigating the irreversible damage evolution processes and fracture mechanisms occurring when materials are subjected to mechanical loading. In this study, NESM is used for the analysis of the acoustic emission (AE) events recorded when marble and cement mortar specimens were subjected to mechanical loading until fracture. In total, AE data originating from four distinct loading protocols are presented. The cumulative distribution of inter-event times (time interval between two consecutive AE events) and the inter-event distances (three-dimensional Euclidian distance between the centers of successive AE events) were examined under the above concept and it was found that NESM is suitable to detect criticality under the terms of mechanical status of a material. This was conducted by evaluating the fitting results of the q-exponential function and the corresponding q-indices of Tsallis entropy $q_{\delta \tau }$ and $q_{\delta r}$, along with the parameters $\tau_{\delta \tau }$ and $d_{\delta r}$. Results support that $q_{\delta \tau }+q_{\delta r}\approx 2$ for AE data recorded from marble and cement mortar specimens of this work, which is in good agreement with the conjecture previously found in seismological data and AE data recorded from Basalt specimens.

## 1. Introduction

Assessing the accumulated damage and understanding the physical mechanisms behind fractures, which lead to critical failures in mechanically loaded specimens or structures, is important for the scientific and engineering community working in situ. For this purpose, many non-destructive testing techniques have been proposed and tested. Among them is the acoustic emission (AE) technique, which deals with the detection of the transient elastic waves generated during the creation and propagation of microcracks in the bulk of the material when it is subjected to mechanical loading. The elastic waves travel within the material towards its boundaries, where they are detected by piezoelectric sensors properly attached on the material’s surface. Acoustic activity data can provide information about the crack evolution processes that take place inside the material [1]. In this context, specific AE related parameters have been used as pre-failure indicators, allowing the estimation of the overall accumulated damage and remaining loading carrying capacity of specimens or full-scale structures [2,3,4,5,6,7]. The AE technique has also been used in controlled laboratory-scale fracture experiments on brittle rocks in order to investigate potential similarities between the fracture mechanisms taking place in mechanically loaded rocks and earthquakes [8]. Acoustic activity and seismic wave propagation can be studied under the same concepts since they both originate from fracture phenomena that are either limited in laboratory scale or extended in field scale. It is generally accepted that both constitute non-linear processes of complex dynamical systems under non-equilibrium states exhibiting multi-fractal and self-similar structure, long range interaction and memory effects [9,10,11,12]. Due to the complexity of these phenomena, the use of advanced statistical analysis methods is favoured instead of classical statistical physics. In this direction, non-extensive statistical physics (NESP), pioneered by Tsallis [13,14,15,16,17], is based on the principle of entropy, and offers a robust analytical method for describing such complex systems. 

Abe and Suzuki 2003 and 2005 [18,19] studied earthquake time and space distances using NESP. They analyzed seismic data from Japan and southern California and found that the cumulative distributions of the three-dimensional Euclidean distances and the time interval between consecutive earthquakes obey the modified Zipf-Mandelbrot law [20], characterized by the q-exponential distributions with $q=q_{\delta r}<1$ [18] and $q=q_{\delta \tau }>1$ [19], respectively. Furthermore, they estimated that $q_{\delta \tau }+q_{\delta r}\approx 2$, where $q_{\delta \tau }$ and $q_{\delta r}$, represent the calculated values of the q-parameter for the temporal and spatial distributions, respectively [18]. This relation was verified by Darooneh and Dadashinia, 2008 [21], where seismic data from Iran were analyzed, and later was reproduced numerically by Hasumi [22] using the two-dimensional Burridge-Knopoff model [23]. Generalized seismicity modeling supporting the dominance of thermodynamics and specifically NESP on a plate-tectonic level is attempted in [24,25,26,27,28,29].

In order to attempt better study of mega-fractures from the large field scale of an earthquake activated region, several works focusing on the small-scale environment of a laboratory were published. Specifically, in order to study the fracture mechanisms taking place in brittle materials such as rocks, laboratory experiments where specimens are subjected to mechanical load have been conducted. The crack evolution processes are recorded using non-destructive monitoring techniques, such as the AE technique [30,31,32,33,34,35] and has been employed successfully in terms of NESP, most notably the studies conducted by Vallianatos et al. [30], Saltas et al. [34], and Greco et al. [35]. In Vallianatos et al. [30] basalt specimens from Mount Etna were subjected to uniaxial compressive loading until fracture and AE data were recorded. The NESP analysis of the acoustic activity showed that the cumulative distributions of the AEs’ scalar moment, the Euclidean distances, and the time-intervals between successive AΕ events are characterized by the q-exponential distributions. Authors also noticed that the sum of the estimated entropic q-indices the time-intervals between two successive AΕ events ($q_{\delta \tau }$) and the Euclidean distances ($q_{\delta r}$) equals to 2 and thus generalizing the estimation that $q_{\delta \tau }+q_{\delta r}\approx 2$ [16,17], for the case of AEs in basalt specimens. Saltas et al. [34] studied the variability of the entropic q-indices and the corresponding $\beta_{q}$ parameters of the recorded AE events when sandstone and marble specimens were subjected to uniaxial compression until fracture. The NESP analysis of the recorded AE data, showed that the cumulative distribution of the inter-event times between successive AΕ events in both cases of the studied materials obeys the q-exponential function. Greco et al. [35] present experimental results concerning acoustic emission (AE) recorded during cyclic compression tests on two different kinds of brittle building materials, namely concrete and basalt. The AE inter-event times were investigated through a non-extensive statistical mechanics (NESM) analysis which shows that their complementary cumulative probability distributions follow q-exponential laws with the entropic index q and the relaxation parameter $\beta_{q}$ to exhibit systematic changes during the various stages of the failure process.

Here, NESM is used for the analysis of AE data recorded when Greek Dionysos marble specimens [36,37] and cement mortar specimens based on Ordinary Portland Cement (OPC) [38] were subjected to mechanical loading until fracture. Specifically, the cumulative distribution of the inter-event distances (i.e., the three-dimensional Euclidian distance between the epicentres of successive AE events) and inter-event times (i.e., the time interval between two consecutive AE events) were examined for successive groups of AE events, until the catastrophic fracture of the specimens and found to obey a q-exponential distribution. Additionally, the corresponding q-entropic indices were calculated, along with the parameters $\tau_{\delta \tau }$ and $d_{\delta r}$. Finally, it is for first time experimentally verified in a small scale laboratory environment the validity of the relation $q_{\delta \tau }+q_{\delta r}\approx 2$ of the calculated entropic q-indices.

## 2. Theoretical Background

The expression of non-extensive Tsallis entropy $S_{q}$, for the case of a variable X (cf. in the case the current work), where X represents a fundamental AE parameter such as the inter-event time δτ and the inter-location δr of two successive AE events with probability distribution function (PDF) p(X) is defined as [13,14,15,17]:

(1) $$S_{q}=\frac{k_{B}}{q-1}\left(1-\sum_{i=1}^{w}p_{i}^{q}\right)$$

where $k_{B}$ is Boltzmann’s constant, w is the total number of microstates, $p_{i}$ is a set of probabilities and q is the entropic index that expresses the degree of non-additivity in a physical system. [13,14,15,16,17].

Tsallis entropy ($S_{q}$) shares many common properties with the standard Boltzmann-Gibbs entropy ($S_{BG}$). However, while $S_{BG}$ is additive and exhibits only short-range correlations, and the total entropy depends on the size of the systems’ subsystems and total microstates, whereas $S_{q}$ (for q≠1) is non-additive, exhibits all-length scale correlations and seems more suitable for complex dynamical systems [13]. Furthermore, the Boltzmann-Gibbs entropy formulation can be recovered by Equation (1) when q≈1: $S_{BG}=-k_{B}\sum_{i=1}^{w}p_{i}\ln p_{i}$. The cases with q>1 and q<1 correspond to sub-additivity and super-additivity, respectively.

For a given system consisting of two statistically independent sub-systems called A and B, the simple additivity of the BG entropy is breached, and the Tsallis entropy satisfies the following expression which describes the non-additive behavior of the system:

(2) $$S_{q}\left(A+B\right)=S_{q}\left(A\right)+S_{q}\left(B\right)+\frac{1-q}{k_{B}}S_{q}\left(A\right)S_{q}\left(B\right)$$

The last term on the right side of Equation (2) indicates the cause of non-additivity of the system (cf. in our case the specimens under severe mechanical loading), and represents the dependency or long-range interactions between microfractures caused by the AE events. Based on Equation (2) the following relations are obtained, for a super-additive (Equation (3)) and a sub-additive system (Equation (4)), respectively [15]:

(3) $$S_{q}\left(A+B\right)>S_{q}\left(A\right)+S_{q}\left(B\right)$$

(4) $$S_{q}\left(A+B\right)<S_{q}\left(A\right)+S_{q}\left(B\right)$$

In order to calculate the probability distribution p(X) of the acoustic parameter X, the non-extensive entropy Tsallis is maximized, using the Lagrange-multipliers method [13,16]. As appropriate parameters for optimizing the non-extensive entropy, the normalization condition and a generalised q-expectation value are used. The normalization condition of p(X) is defined as:

(5) $$\int_{0}^{\infty }p(X)dX=1$$

The generalized q-expectation value, *X_q_*, is given by:

(6) $$X_{q}=\langle X_{q}\rangle =\int_{0}^{\infty }XP_{q}\left(X\right)dX=1$$

where the escort probability $P_{q}\left(X\right)$ is defined in [16] as:

(7) $$P_{q}\left(X\right)=\frac{P^{q}\left(X\right)}{\int_{0}^{\infty }P^{q}\left(X\right)dX^{\prime }}$$

The maximization of Tsallis entropy $S_{q}$ under consideration of Equations (5) and (6), leads to the probability density function below [13,16]:

(8) $$p\left(X\right)=\frac{{\left[1-\left(1-q\right)\beta_{q}X\right]}^{1/\left(1-q\right)}}{Z_{q}}=\frac{\exp_{q}\left(-\beta_{q}X\right)}{Z_{q}}$$

where *Z_q_* refers to the q partition function: $Z_{q}=\int_{0}^{X\max}\exp_{q}\left(-\beta_{q}X\right)dX$. The entropic quantity $\beta_{q}$ is defined as: $\beta_{q}=\beta^{*}/\left(c_{q}+\left(1-q\right)\beta X_{q}\right)$ where, $\beta^{*}$ represents a Lagrange multiplier and $c_{q}={\int_{0}^{X_{\max}}\left[p\left(X\right)\right]}^{q}dX$. The term $\exp_{q}\left(X\right)$ denotes the q-exponential function defined by:

(9) $$\exp_{q}\left(X\right)=\{\begin{array}{cc} {\left[1+\left(1-q\right)X\right]}^{\frac{1}{1-q}} & \begin{array}{cc} \mathrm{for} & \left[1+\left(1-q\right)X\right]\geq 0 \end{array}\\ 0 & \begin{array}{cc} \mathrm{for} & \left[1+\left(1-q\right)X\right]<0 \end{array} \end{array}$$

The inverse form of Equation (9) is called q-logarithmic function and is given by:

(10) $$\ln_{q}\left(X\right)=\frac{1}{1-q}\left(X^{1-q}-1\right)$$

In the case when q≈1 both Equations (9) and (10) correspond to the ordinary exponential and logarithmic function, respectively. In the case when q>1 a tail of power law form appears, whereas in the case of 0<q<1 the q-exponential function presents a cut-off [18,19].

In non-extensive statistical physics, the quantity to be compared with the distribution of the observed system is not the original p(X) but its associated escort distribution $P_{q}\left(X\right)$ [18,19,39]. The normalized cumulative distribution of the acoustic parameter X, expressed as a q-exponential function, is obtained by integrating the probability density function p(X):

(11) $$P\left(>X\right)=\int_{X}^{\infty }P_{q}\left(X\right)dX=\exp_{q}\left(-\frac{X}{\left(1-q\right)\langle X_{q}\rangle +\frac{1}{\beta *}}\right)$$

The last right-hand term of Equation (11) suggests that after the estimation of the appropriate q, which describes the distribution of the acoustic parameter X, the logarithmic function lnq[P(>X)] calculated by basic algebra rules $\ln_{q}\left[P\left(>X\right)\right]=-{\left[\left(1-q\right)\langle X_{q}\rangle +\frac{1}{\beta *}\right]}^{-1}X$, is linear in accordance to X with slope $l=-\frac{1}{\left(1-q\right)\langle X_{q}\rangle +\frac{1}{\beta *}}$.

In the context of the present work, the continuous variable X represents the AE parameters of inter-event time δτ and inter-event distance δr. Thus, the AE timeseries is transformed, for the case of parameter δτ, to the inter-event time timeseries $\delta \tau_{i}=t_{i+1}-t_{i}$, for the case of parameter δr, to the Euclidean distance time series $\delta r_{i}=\sqrt{\Delta x_{i}{}^{2}+\Delta y_{i}{}^{2}+\Delta z_{i}{}^{2}}$. NESM was applied to the newly formed timeseries and in each case the normalized cumulative distribution of inter-event times P(>X) was plotted, the AE data were fitted with a q-exponential function and the corresponding q indices, along with the parameters $\tau_{\delta \tau }$ and $d_{\delta r}$, were calculated.

## 3. Specimens and Experimental Protocol

For the needs of the current work, four experimental protocols were conducted, i.e., uniaxial compression, direct tension, three-point bending and shear, during which both mechanical and AE data were recorded. The specimens used during the conducted experiments were Greek Dionysos marble and cement mortar based on Ordinary Portland Cement. Dionysos marble is used widely in the restoration projects of the Athenian Acropolis monuments, due to the fact that it presents the same composition and properties as the original building stone of the monuments [4,36,37,40]. Ordinary Portland Cement is the most commonly used type of cement masonry and is extensively used in the construction industry [38], as the understanding of the fracture mechanisms of cement-based materials is of a great importance for the safety of both infrastructure and humans. The cement mortar specimens preparation process and recipe is described in previous works [41,42]. In total, four marble and three cement mortar specimens are chosen to be presented here.

Depending on the adopted experimental protocol, the specimens’ material and individual geometries and characteristics the experiments are categorised using the coding A to E, as can be seen in Table 1. The right most column shows the total recorded Acoustic Emissions (AE) events of each specimen. More specifically:

Experiment A includes prismatic marble specimens with dimensions of 45 × 45 × 100 mm^3^. The specimens were subjected to uniaxial compressive stress under quasi-static conditions and load control mode at a constant rate of 0.2MPa/s. In order to monitor the acoustic activity and estimate the location of the AE events, four R16α acoustic sensors were properly attached on the specimens’ surface. More details regarding the experimental set-up can be found in [43,44].

Experiment B comprises of notched beam-shaped marble specimens subjected to three-point bending under quasi-static conditions and displacement-control mode at a rate of 0.01 mm/min. The dimensions of the specimen that was chosen to be presented here were 25 × 25 × 100 mm^3^ with a notch of 2.5 mm width and 6 mm length located at the midspan of the beam. Four R16α acoustic sensors were properly attached on the specimens’ surface in order to monitor the location of the AE events [45].

Experiment C includes marble double edge notched specimens of dog-bone shape, subjected to direct tension under quasi-static conditions and displacement-control mode at a rate of 0.2 mm/min. The dimensions of the notches of the specimen presented in this paper were equal to 3 mm width and 8 cm length. Six R16α acoustic sensors were attached to the specimen’s surface, in an attempt estimate the location of the acoustic events. Further information regarding the geometry of the specimen and the experimental apparatus used for the implementation of the experimental protocol can be found in [46,47].

Experiment D refers to a complex structure of two independent marble blocks, a prismatic one with dimensions of 25 × 25 × 20 cm^3^ and a “Γ”-shaped one. The two marble blocks were connected with an “I”-shaped titanium connector and cement-based filling. The structure was subjected to shear loading under quasi-static conditions with displacement control mode at a constant rate equal to 0.2 mm/min. Eight R15α AE sensor, were properly attached on the specimen’s surface in order to monitor and determine the location of the acoustic events. Further experimental details can be found in [48].

Three experiments coded as E were performed on beam-shaped cement mortar specimens with square cross-section of 150 × 150 mm^2^ and length of 700 mm, with a notch of 5 mm depth and 5 mm width located at their midspan. Depending on the existence and nature of reinforcement are labelled as follows: (i) without internal reinforcement (experiment E-1), (ii) with short steel fibres in 25 kg/m^3^ ratio (experiment E-2), and (iii) with short plastic fibres, in 4 kg/m^3^ ratio (experiment E-3). The specimens were subjected to three-point bending under quasi-static conditions and displacement-control at a rate equal to 0.08 mm/min. The acoustic activity was monitored with the aid of eight acoustic sensors of the R15α type, attached on the specimens’ surface. Additional information regarding the implemented experimental protocol can be found in [49].

The experiments were conducted using an MTS-insight loading frame of capacity 10 kN, in the case of experiments A to D and an Instron 1126 loading frame of capacity 600 kN for the case of experiments E. Table 1 summarises the details regarding the loading protocols, the materials and the total number of the AE events recorded during the individual experiments presented above.

## 4. Results and Discussion

Based on Equation (11), the normalized cumulative distribution functions (CDFs) of the AE inter-event times P(>δτ) and the AE inter-event distances P(>δr), have been found [30,34] to obey a q-exponential function given by the Equations (12) and (13):

(12) $$P\left(>\delta \tau \right)=\exp_{q}\left(\frac{\delta \tau }{\tau_{\delta \tau }}\right)={\left[1+\left(q_{\delta \tau }-1\right)\frac{1}{\tau_{\delta \tau }}\delta \tau \right]}^{\frac{1}{1-q_{\delta \tau }}}$$

(13) $$P\left(>\delta r\right)=\exp_{q_{\delta r}}\left(\frac{\delta r}{d_{\delta r}}\right)={\left[1+\left(q_{\delta r}-1\right)\frac{1}{d_{\delta r}}\delta r\right]}^{\frac{1}{1-q_{\delta r}}}$$

where the parameters $\tau_{\delta \tau }$ and $d_{\delta r}$ express a q relaxation property. It should be stressed that the probability density function P(>X) of the q-exponential distribution given by Equation (11) and the normalized cumulative distribution functions of the AE inter-event times P(>δτ) and the AE inter-event distances P(>δr) expressed by the Equations (12) and (13) exhibit the same mathematical formulation. For the conducted experiments, the q-entropic indices $q_{\delta \tau }$ and $q_{\delta r}$ along with their corresponding parameters $\tau_{\delta \tau }$ and $d_{\delta r}$ were calculated for each fitting curve and are presented in Table 2.

**Figure 1:**
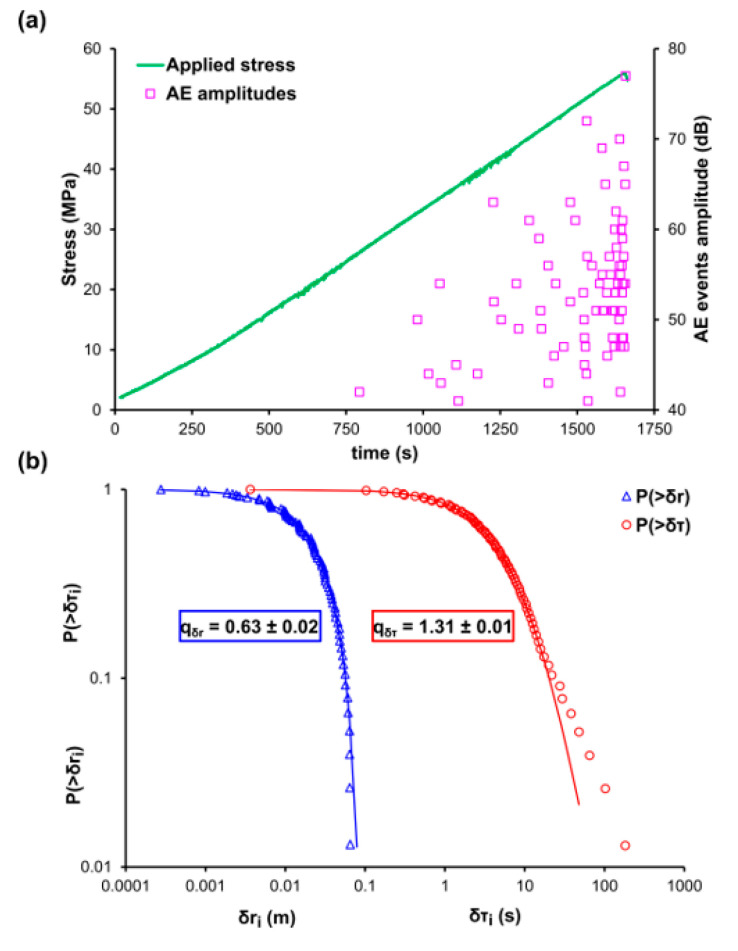
(**a**) The temporal variation of the applied mechanical stress (green line) and the amplitudes of the recorded AE events (pink square markers) for the case of the marble specimen of the experiment A. (**b**) The cumulative distribution function (CDF) of the AE inter-event times (red circle markers) and inter-event distances (blue triangle markers), along with the corresponding q-exponential fitting curves (red and blue solid curves).

Figure 1a shows the temporal evolution of the applied mechanical stress (green line) and the corresponding amplitudes of the recorded AE events (pink square markers) during the experimental procedure of compressional tests (experiment A specimens). It is worthy to stress that, the majority of the AE are recorded above the 60% of the uniaxial compressive strength of the loading and well before the critical fracture of the specimen, when the fracture mechanisms are more prominent and the microcracks have started to congregate into macrocracks. Figure 1b shows the log-log plot of the cumulative distributions of the AE inter-event times P(>δτ) (red circle markers) and AE inter-event distances P(>δr) (blue triangle markers), and their corresponding q-indices which have been calculated through the Equations (12) and (13), respectively, and found to be equal to $q_{\delta \tau }\approx 0.63$ and $q_{\delta r}\approx 1.38$. Focusing on long times δτ and long distances δr of Figure 1b of both CDFs it becomes clear that the fitting points deviate from the corresponding modelled ones, and that such a behaviour can be attributed to the limitation of the modelling and the degree of selected subsystems. It should be noted that the data points located at the tail region of both CDFs amount for the 8% of the total points of the AE inter-event times P(>δτ) and 4% of the total data of the AE inter-event distances P(>δr), in order to avoid possible bias during the q-indices calculations, the tail region is excluded and only the data points from the first and the middle region of the CDFs were used. Table 3 shows the percentage of the experimental data of both CDFs, which are located at the tails region and deviated from the corresponding NESM modelling.

**Figure 2:**
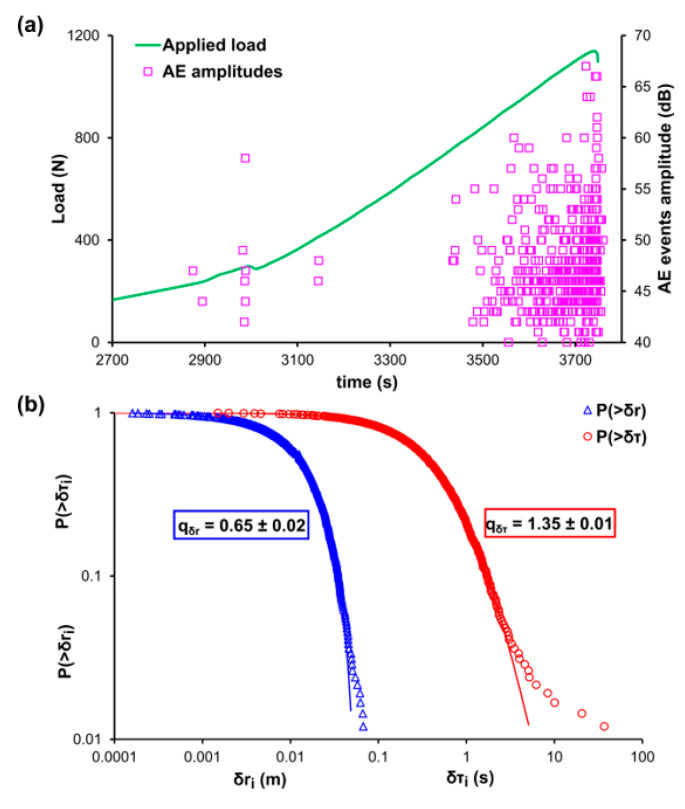
(**a**) The temporal variation of the applied mechanical load (green line) and the amplitudes of the recorded AE events (pink square markers) for the case of the marble specimen during the three-point bending experiment B. (**b**) The cumulative distribution function (CDF) of the AE inter-event times (red circle markers) and inter-event distances (blue triangle markers), along with the corresponding q-exponential fitting curves (red and blue solid curves).

The experimental recordings and the corresponding analyses for the three-point bending tests of experiment B specimens are shown in Figure 2a,b. Figure 2a shows the temporal evolution of the applied mechanical load (green line) and the corresponding distribution of the AE events (pink square markers), while Figure 2b depicts the CDFs for the AE inter-event times P(>δτ) (red circle markers) and AE inter-event distances P(>δr) (blue triangle markers) in a log-log plot. Considering that experiment B refers to three-point bending loading protocol and the size of the used specimens, it is expected to observe low bending strength (i.e., 1.1 kN approximately). Figure 2a, is plotted for the last 1000 s of the experimental procedure. It is noted that before that second, no AE activity was recorded. This is due to the fact that during bending of brittle geomaterials the damages initiate at very low load levels and fractures rapidly occur due to the low tensional strength of the specimen’s lower side. In order to avoid possible bias, the q-indices have been calculated excluding the data points from the tail region of both CDFs using Equations (12) and (13) and were found to be $q_{\delta \tau }\approx 0.65$ and $q_{\delta r}\approx 1.35$, respectively. The data points belonging to the tail regions of both CDFs correspond to the 3% of the total points of the AE inter-event times P(>δτ) and 2% of the total data of the AE inter-event distances P(>δr).

**Figure 3:**
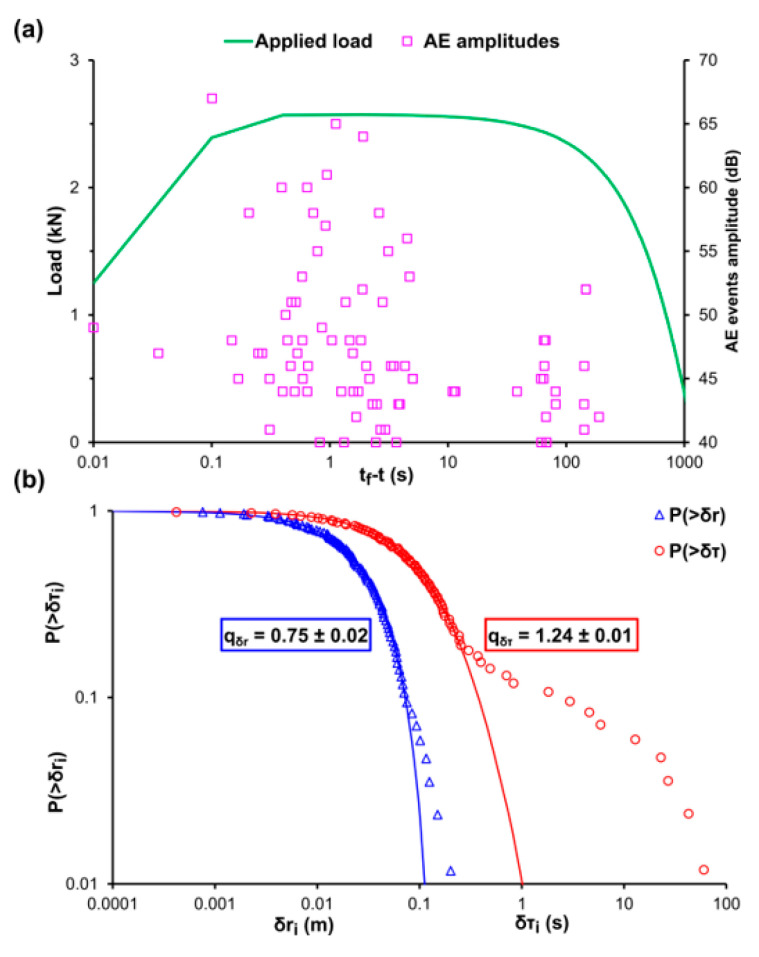
(**a**) The temporal variation, in terms of the logarithmic (t_f_-t) scale [50], of the applied mechanical load (green line) and the amplitudes of the recorded AE events (pink square markers) for the case of the marble specimen during the direct tension experiment C. (**b**) The cumulative distribution function (CDF) of the AE inter-event times (red circle markers) and inter-event distances (blue triangle markers), along with the corresponding q-exponential fitting curves (red and blue solid curves).

Figure 3a shows the temporal recording of the applied mechanical load (green line) and the recorded amplitudes of the AE events (pink square markers), for the experimental protocol of direct tension and the specimens of experiment C, using for time scale the time to failure parameter $\left(t_{f}-t\right)$, with $t_{f}$ being the moment when the critical fracture of the specimen occurred. It should be noted that due to the nature of the conducted experimental protocol (i.e., direct tension) during which no acoustic activity is recorded, apart from some isolated AE events during the early stages of the loading protocol, until the very last seconds before the fracture of the specimen, the usage of the $\left(t_{f}-t\right)$ parameter was preferred since it provides a better understanding regarding the acoustic activity during the last stages of the loading protocol and the dynamic processes occurring in the bulk of the specimen, leading it to criticality and ultimately to fracture [50]. Furthermore, considering that experiment C refers to a marble specimen subjected to direct tension, it is reasonable to observe low tensional strength values with the ultimate tensional strength of the specimen recorded at approximately 2.6 kN. Figure 3b shows the log-log plot of the CDFs of the AE inter-event times P(>δτ) (red circle markers) and AE inter-event distances P(>δr) (blue triangle markers), along with the corresponding q-indices that have been calculated via Equations (12) and (13) using data points from the first and middle area of the CDFs, equalling to $q_{\delta \tau }\approx 0.75$ and $q_{\delta r}\approx 1.24$. Notice the deviation of the fitting points from the experimental ones at the tails of both CDFs which correspond to long times δτ and long distances δr when dynamic processes take place, a phenomenon which can be attributed to the microcrack formation processes occurring inside the material during the early stages of the loading protocol. The appearance of long times δτ, during the early stages of the loading protocol, is in agreement with the recorded AE amplitudes of Figure 3a where we notice a premature concentration of AE events, around the 100 s of the $\left(t_{f}-t\right)$ time scale, before the main acoustic activity around the 1 s of the $\left(t_{f}-t\right)$ time scale, which leads to the fracture of the marble specimen. It should be mentioned that the data points of the tail region of both CDFs refer 15% of the total points of the AE inter-event times P(>δτ) and 6% of the total data of the AE inter-event distances P(>δr).

**Figure 4:**
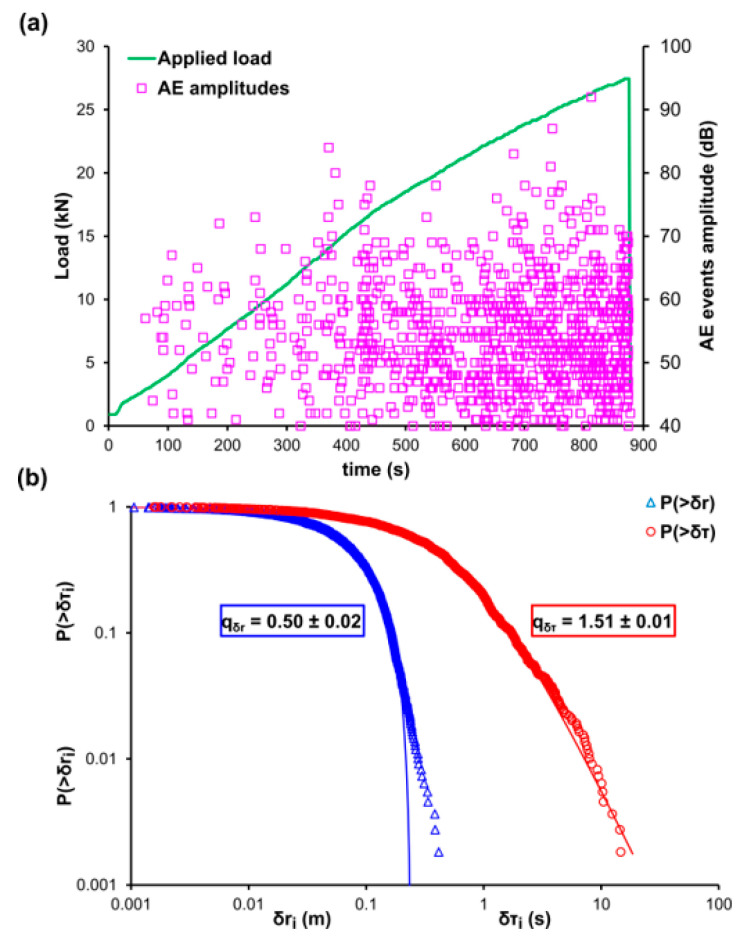
(**a**) The temporal variation of the applied mechanical load (green line) and the amplitudes of the recorded AE events (pink square markers) for the case of the marble specimen during the shear experiment D. (**b**) The cumulative distribution function (CDF) of the AE inter-event times (red circle markers) and inter-event distances (blue triangle markers), along with the corresponding q-exponential fitting curves (red and blue solid curves).

The marble specimen of experiment D was subjected to shear loading. Figure 4a shows temporal variation of the applied mechanical load (green line) and the amplitudes of the recorded AE events (pink square markers). Notice, the abundance of AE events throughout the duration of the loading protocol. It is worth mentioning that the high number of AE events recorded throughout the duration of the loading protocol is due the complexity and dimensions of the specimen being subjected to shear loading. It is also observed the high loading values with a fracturing value of 27.5 kN. Figure 4b shows the log-log plot of the CDFs of the AE inter-event times P(>δτ) (red circle markers), the inter-event distances P(>δr) (blue triangle markers), and their corresponding q-indices, $q_{\delta \tau }\approx 0.50$ and $q_{\delta r}\approx 1.51$, that have been calculated using Equations (12) and (13), respectively. In this case, there are no strong deviations at the tails of both CDFs from the fitting points (solid blue and red lines) comparing to the previous experimental protocols, however, the calculation of the q-indices was performed using data points from the first and middle area of the CDFs. The data points belonging to the tail regions of both CDFs correspond to the 4% of the total points of the AE inter-event times P(>δτ) and 4% of the total data of the AE inter-event distances P(>δr).

**Figure 5:**
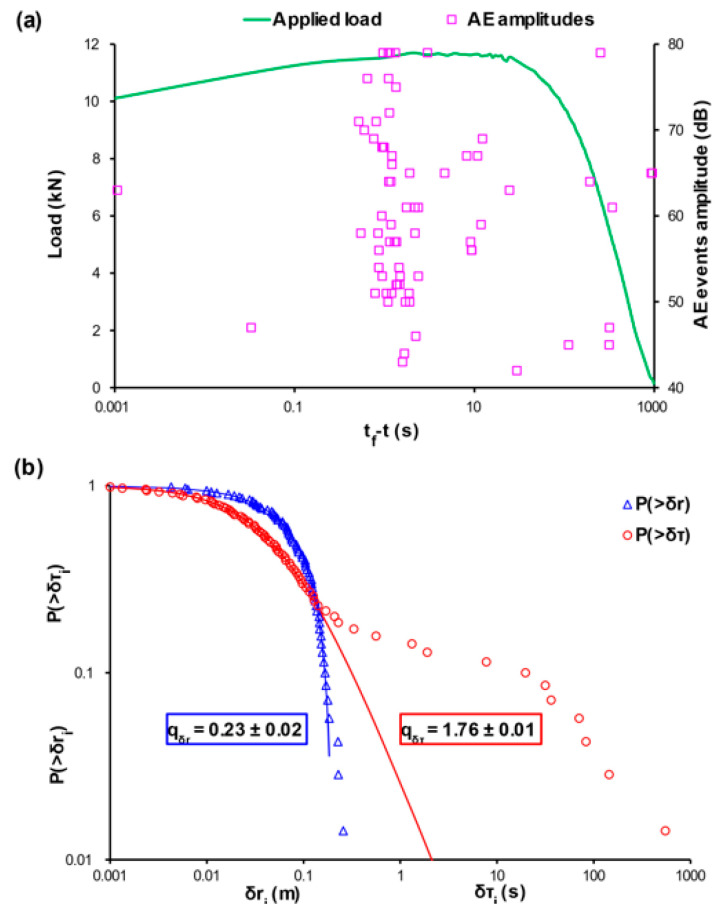
(**a**) The temporal variation, in terms of the logarithmic (t_f_-t) scale [50], of the applied mechanical load (green line) and the amplitudes of the recorded AE events (pink square markers) for the case of the cement specimen during the three-point bending experiment E1 with no internal reinforcement, in terms of the logarithmic(tf-t) scale [50]. (**b**) The cumulative distribution function (CDF) of the AE inter-event times (red circle markers) and inter-event distances (blue triangle markers), along with the corresponding q-exponential fitting curves (red and blue solid curves).

**Figure 6:**
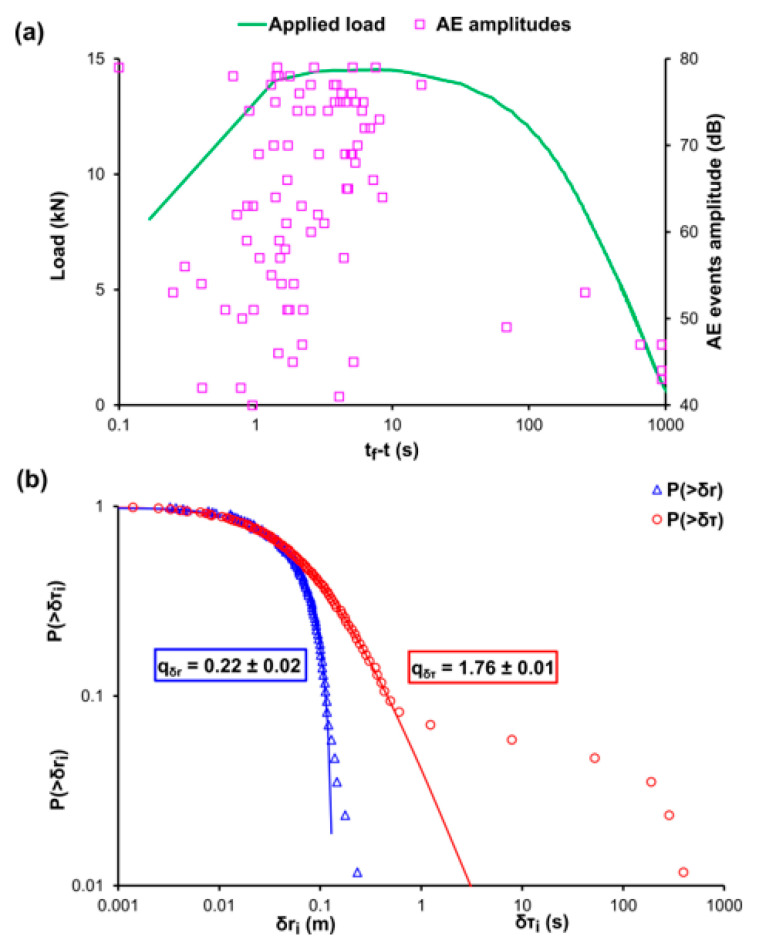
(**a**) The temporal variation, in terms of the logarithmic (t_f_-t) scale [50], of the applied mechanical load (green line) and the amplitudes of the recorded AE events (pink square markers) for the case of the cement specimen during the three-point bending experiment E-2 embedded with steel fibres as internal reinforcement, in terms of the logarithmic (t_f_-t) scale [50]. (**b**) The cumulative distribution function (CDF) of the AE inter-event times (red circle markers) and inter-event distances (blue triangle markers), along with the corresponding q-exponential fitting curves (red and blue solid curves).

**Figure 7:**
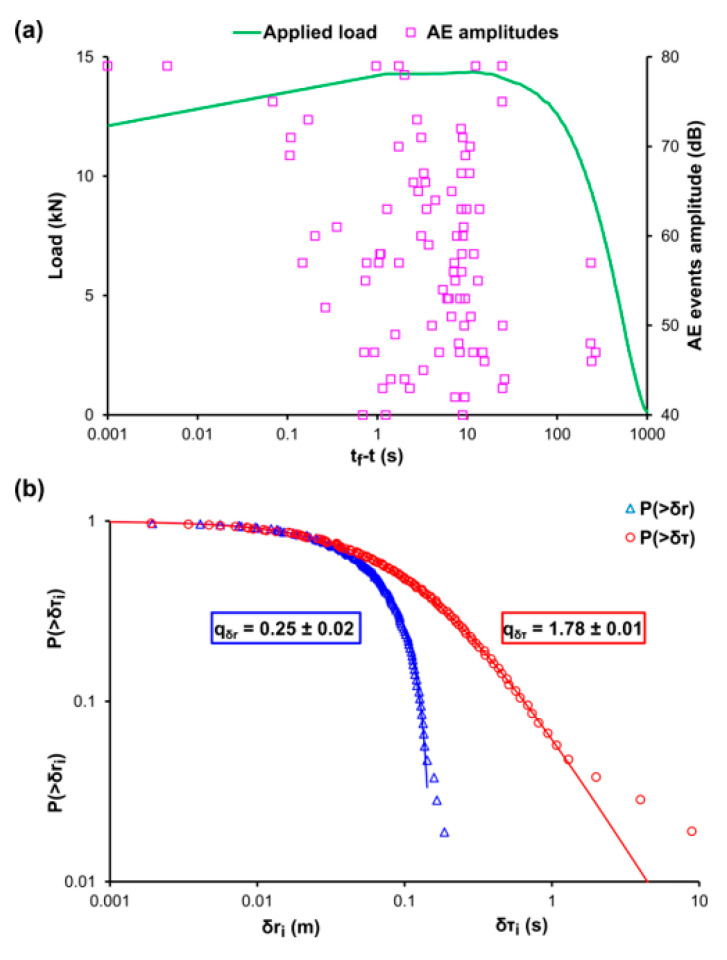
(**a**) The temporal variation, in terms of the logarithmic (t_f_-t) scale [50], of the applied mechanical load (green line) and the amplitudes of the recorded AE events (pink square markers) for the case of the cement specimen during the three-point bending experiment E-3 embedded with plastic fibres as internal reinforcement, in terms of the logarithmic (t_f_-t) scale [50]. (**b**) The cumulative distribution function (CDF) of the AE inter-event times (red circle markers) and inter-event distances (blue triangle markers), along with the corresponding q-exponential fitting curves (red and blue solid curves).

Figure 5a, Figure 6a, and Figure 7a, depict the temporal variation of the applied mechanical load (green line) and the amplitudes of the recorded AE events (pink square markers) using the time to failure parameter $\left(t_{f}-t\right)$ scale for the cases of the cement mortar specimens that were subjected to three-point bending with different levels of internal reinforcement, labelled E-1, E-2, and E-3, respectively. Since experiments E-1, E-2, and E-3 specimens refer to three-point bending and considering the low flexural strength of the cement, the acoustic activity is recorded in the last stages of the loading protocol. Therefore, the use of the $\left(t_{f}-t\right)$ scale was decided. Figure 5b, Figure 6b, and Figure 7b present the CDFs of the AE inter-event times P(>δτ) (red circle markers) and AE inter-event distances P(>δr) (blue triangle markers), as well as their corresponding q-indices $q_{\delta \tau }$ and $q_{\delta r}$ which were calculated through fitting, based on Equations (12) and (13) using all the data points excluding those in the tails of the CDFs. Regarding the specimen of experiment E1, with no internal reinforcement the q-indices were found to be equal with $q_{\delta \tau }\approx 0.23$ and $q_{\delta r}\approx 1.76$, while for the specimen of experiment E-2, i.e., the one with the steel fibres acting as internal reinforcement, were equal to $q_{\delta \tau }\approx 0.22$ and $q_{\delta r}\approx 1.76$, lastly, for the specimen of experiment E-3 in which plastic fibres are implemented, the q indices were $q_{\delta \tau }\approx 0.25$ and $q_{\delta r}\approx 1.78$. In the case of the specimen of experiment E-1 in which there is no internal reinforcement, it is observed a sharp deviation from the points of the NESM modelling (red solid line) in the case of the long times δτ, which amount roughly for the 19% of the total experimental points, in contrast to the δr model (solid blue line) which satisfactorily describes the values of the calculated data points, with only 4% of the total experimental data being located to the tail region of the CDF. The deviation at the tail region of the CDF of the AE inter-event times P(>δτ) is in accordance with the distribution of the recorded AE events of Figure 5a, where during the early stages of the loading protocol, spanning between 10 s and 1000 s of the $\left(t_{f}-t\right)$ scale an early acoustic activity comprising of occasional AE events appears. The tail region refers to the 15% of the total experimental data. A similar behaviour is shown in the specimen of experiment E-2, which is reinforced with steel fibres, where in Figure 6a an early concertation of AE events, ranging from 100 s to 1000 s of the $\left(t_{f}-t\right)$ scale, is recorded. It is worth mentioning the data points belonging to the tail regions of both CDFs correspond to the 7% of the total points of the AE inter-event times P(>δτ) and 6% of the total data of the AE inter-event distances P(>δr). In contrast, the specimen of experiment E-3, which has plastic fibres as internal reinforcement, does not show great deviations from the models, with the data points being located to the tail regions of both CDFs amounting for the 3% of the total data of the AE inter-event times P(>δτ) and 3% of the total data of the AE inter-event distances P(>δr). These behaviors are expected considering the fact that the cement is a very brittle material with low bending strength, meaning that the fracture mechanisms will be firstly activated at low values of the applied mechanical load, expressed as early AE events with long inter-event times δτ between them, hence the discrepancy in Figure 5b and Figure 6b in the case of the CDFs of the AE inter-event times P(>δτ). We note that this observed pattern is in similarity with that observed in Figure 3 of Bogachev et al. [51] supporting the view of the universality of Tsallis Statistics. Furthermore, the reinforcement with plastic fibers increased the bending strength of the cement specimen of experiment E-3, in relation to the unreinforced specimen of experiment E-1 and the specimen of experiment E-2 (steel fibers), resulting in fewer AE events during the early loading stages.

## 5. Conclusions

In this work, NESM analyses were applied to marble and cement mortar specimens that were subjected to mechanical loading of various protocols until fracture. The specimens were subjected to four distinct experimental protocols, i.e., uniaxial compression, direct tension, three-point bending and shear. During the experiments, loading and AE data were recorded and the normalized CDFs of the inter-event times P(≥δτ) and inter-event distances P(≥δr) of the entire AE time series were plotted and fitted using exponential NESM modeling. The entropic q-indices $q_{\delta \tau }$ and $q_{\delta r}$, along with the parameters $\tau_{\delta \tau }$ and $d_{\delta r}$ were calculated, according to the Tsallis entropy model, for each specimen. The fitting results manifest that the entropic index $q_{\delta \tau }$, which describes the AE inter-event time distribution for all the cases of specimens, is always $q_{\delta \tau }>1$, thus, this process is dominated by NESM and can be characterized as a super-additive process while, the entropic index $q_{\delta r}$ which is associated with the AE inter-event Euclidian distances, is expressed by the NESM of a sub-additive process since it is always $q_{\delta r}<1$. Conclusively, the sum of the calculated values of the q-parameters for the spatial and temporal distributions, $q_{\delta \tau }$ and $q_{\delta r}$ was found for every specimen. Results clearly verify and generalize the duality of both entropic indices based on the relation that $q_{\delta \tau }+q_{\delta r}\approx 2$, in the case of AE event in marble and cement mortar specimens, similar to the case of the AE events in Basalt specimens [30] and seismicity [18,21,22,27].

## Figures and Tables

**Table 1:** The experimental details of the presented specimens.

Experiment	Loading Protocol	Material	Total AE Events
A	Uniaxial compressive stress	Marble	78
B	Three-point bending	“	418
C	Direct tension	“	85
D	Shear	“	1095
E-1	Three-point bending	Cement mortar	70
E-2	“	“	86
E-3	“	“	105

“: This is a ditto mark indicating that the words above it in the table are to be repeated.

**Table 2:** The calculated values of the entropic indices $q_{\delta \tau }$ and $q_{\delta r}$, along with the calculated fitting parameters $\tau_{\delta \tau }$ and $d_{\delta r}$, for the presented specimens. The last right column shows the sum of both entropic indices.

Experiment	Inter-Event Times		Inter-Event Distances		$q_{\delta \tau }+q_{\delta r}$
	$q_{\delta \tau }$	$\tau_{\delta \tau }\;(s)$	$q_{\delta r}$	$d_{\delta r}\;(m)$	
A	1.38	5.500	0.63	0.036	2.01 ± 0.02
B	1.35	0.487	0.65	0.022	2.00 ± 0.02
C	1.24	0.122	0.75	0.041	1.99 ± 0.02
D	1.51	0.388	0.50	0.121	2.01 ± 0.02
E-1	1.76	0.050	0.23	0.154	1.99 ± 0.02
E-2	1.76	0.073	0.22	0.106	1.98 ± 0.02
E-3	1.78	0.102	0.25	0.116	2.03 ± 0.02

**Table 3:** Percentage of the experimental data of the CDFs of the AE inter-event times *P(>δτ)* and AE inter-event distances *P(>δr)* deviating from the corresponding NESM modelling.

Experiment	Inter-Event Times P(>δτ)	Inter-Event Distances P(>δr)
A	8%	4%
B	3%	2%
C	15%	6%
D	4%	4%
E-1	19%	4%
E-2	7%	6%
E-3	3%	3%
